# Accelerated partial breast irradiation with 3-dimensional conformal and image-guided intensity-modulated radiotherapy following breast conserving surgery – 7-Year results of a phase II trial

**DOI:** 10.1016/j.breast.2020.10.010

**Published:** 2020-10-30

**Authors:** Norbert Mészáros, Tibor Major, Gábor Stelczer, Levente Jánváry, Zoltán Zaka, Dávid Pukancsik, Zoltán Takácsi-Nagy, János Fodor Md, Csaba Polgár

**Affiliations:** a Center of Radiotherapy, National Institute of Oncology, Budapest, Hungary; b Department of Oncology, Semmelweis University, Faculty of Medicine, Budapest, Hungary; c Department of Breast and Sarcoma Surgery, National Institute of Oncology, Budapest, Hungary

**Keywords:** Breast cancer, Phase II trial, Partial breast irradiation, 3-Dimensional conformal radiotherapy, Image-guided intensity-modulated radiotherapy

## Abstract

**Purpose:**

To present the 7-year results of accelerated partial breast irradiation (APBI) using three-dimensional conformal (3D-CRT) and image-guided intensity-modulated radiotherapy (IG-IMRT) following breast-conserving surgery (BCS).

**Patients and methods:**

Between 2006 and 2014, 104 patients were treated with APBI given by means of 3D-CRT using 3–5 non-coplanar, isocentric wedged fields, or IG-IMRT using kV-CBCT. The total dose of APBI was 36.9 Gy (9 × 4.1 Gy) using twice-a-day fractionation. Survival results, side effects and cosmetic results were assessed.

**Results:**

At a median follow-up of 90 months three (2.9%) local recurrences, one (0.9%) regional recurrence and two (1.9%) distant metastases were observed. The 7-year local (LRFS), recurrence free survival was 98.9%. The 7-year disease-free (DFS), metastases free (MFS) and overall survival (OS) was 94.8%, 97.9% and 94.8%, respectively. Late side effects included G1 skin toxicity in 15 (14.4%), G1, G2, and G3 fibrosis in 26 (25%), 3 (2.9%) and 1 (0.9%) patients respectively. Asymptomatic (G1) fat necrosis occurred in 10 (9.6%) patients. No ≥ G2 or higher late side effects occurred with IMRT. The rate of excellent/good and fair/poor cosmetic results was 93.2% and 6.8%, respectively.

**Conclusion:**

7-year results of APBI with 3D-CRT and IG-IMRT are encouraging. Toxicity profile and local tumor control are comparable to other series using multicatheter interstitial brachytherapy. Therefore, these external beam APBI techniques are valid alternatives to whole breast irradiation and brachytherapy based APBI.

## Highlights

• Phase II APBI trial using 3D-CRT or IG-IMRT.
• Twice-a-day fractionation, with a total dose of 36.9 Gy (9 × 4.1Gy).
• No Grade 2 or worst late side effects with IG-IMRT at median follow up of 90 months.
• These APBI techniques are valid alternatives to WBI or brachytherapy based APBI.

## Introduction

Whole breast irradiation (WBI) after breast conserving surgery (BCS) was the standard care of early stage breast cancer patients for decades. Several prospective randomized studies and their meta-analysis demonstrated that WBI improved both local tumour control and survival [[1], [2], [3]]. However, the majority of early ipsilateral breast recurrences occur in the tumour bed or in immediate vicinity, which has been confirmed by numerous studies [[4], [5], [6]]. In the past decades, accelerated partial breast irradiation (APBI) has become an increasingly utilized treatment option for early stage breast cancer patients after BCS, significantly reducing the time and cost of radiotherapy (RT) and the exposure of normal tissues to radiation [[7], [8], [9], [10]]. Various techniques for APBI delivery have been explored, including interstitial (multicatheter) brachytherapy [[9], [10], [11], [12], [13], [14]], single-entry brachytherapy with intracavitary devices (e.g. MammoSite) [15], external beam RT (EBRT) utilizing three-dimensional conformal RT (3D-CRT) [[16], [17], [18], [19], [20], [21], [22], [23]] or image-guided intensity-modulated RT (IG-IMRT) [[24], [25], [26], [27], [28], [29], [30], [31], [32], [33]], protons [34,35], and intraoperative RT (IORT) as well [36,37]. APBI using high-dose-rate interstitial brachytherapy (HDR-iBT) in low-risk patients provided tumour control comparable to WBI resulting in favourable cosmetic, toxicity profile and quality of life effects [[12], [13], [14]]. Several phase II studies [[16], [17], [18], [19], [20], [21], [22], [23]] and the NSABP-B39 phase III clinical trial [38] demonstrated that 3D-CRT provides results comparable to WBI, in terms of local tumour control, early and late side effects and quality of life. Recently, we have also reported encouraging 5-year results obtained by 3D-CRT APBI [18]. Here, we are reporting the 7-year clinical results, side-effects and cosmetic results of our prospective sequential phase II clinical study using 3D-CRT and IG-IMRT for the delivery of APBI.

## Methods and materials

This single institutional, sequential, single-arm phase II study was designed to test the feasibility, safety and efficacy of external beam APBI. Between 2006 and 2011 we treated 44 patients with 3D-CRT, the 5-year results were published in 2014 [18]. In the second part, between 2011 and 2014, - when IG-IMRT became available at our institute-, we enrolled and treated 60 patients with IG-IMRT, the dosimetry and 3-year results were published in 2017 [33]. Primary end-points were late radiation side effects. Secondary end-points included early toxicities, local and regional tumor control, disease-free, overall and cancer-specific survival, quality of life, as well as cosmetic results. According to our primary hypothesis, we considered that APBI with 3D-CRT or IG-IMRT will cause less or an equal rate of severe late toxicity compared with conventional WBI or multicatheter brachytherapy based APBI.

Patients underwent BCS, with at least 2 mm clear resection margins and during the surgery the boundaries of the excision cavity were marked with 4–6 titanium clips. The type of BCS was radioguided occult lesion localisation (ROLL) excision in 17 (16.3%), quadrantectomy in 17 (16.3%) and wide excision in 70 (67.4%) patients, with sentinel lymph node biopsy in 100 (96.1%) and axillary dissection in 4 (3.9%) patients. We reported the eligibility criteria in detail in our previous publications [18,33]. Briefly, patients were eligible for APBI if they were aged 40 years old or older, with unifocal invasive tumor up to a diameter of 3 cm, with negative axillary lymph node status (pN0) and with microscopically clear inked surgical margins by at least 2 mm. We excluded patients if they have multiple tumor foci, invasive tumors with the presence of an extensive intraductal component (EIC); lympho-vascular invasion (LVI), Paget-disease of the nipple, bilateral breast cancer, prior history of breast cancer or other malignant disease within 5 years. Patient and tumor characteristics are listed in Table 1. The study protocol was evaluated and accepted by the institutional and national ethics committees, and all patients provided written informed consent before enrolment. The trial was registered at ClinicalTrials.gov with an identifier number of NCT-02003560.

**Table 1:** Patient, tumor and adjuvant treatment characteristics.

Characteristics	n (%)^a^
Mean age (range)	61.7 ys. (41–77)
Age groups (years)	
≤40	0 (0%)
41–50	7 (6.7%)
51–60	37 (35.6%)
61–70	48 (46.1%)
>70	12 (11.6%)
Postmenopausal	95 (91.3%)
Breast cup size	
A	2 (1.9%)
B	37 (35.6%)
C	47 (45.2%)
D, D+	18 (17.3%)
Laterality	
Right	57 (54.8%)
Left	47 (45.2%)
Tumor location (quadrant)	
Upper-outer	61 (58.6%)
Lower-outer	14 (13.5%)
Upper-inner	15 (14.4%)
Lower-inner	8 (7.7%)
Central	6 (5.8%)
Pathological tumor size (mm)	
≤5	3 (2.9%)
>5-10	40 (38.5%)
>10-20	56 (53.8%)
>20-30	5 (4.8%)
Median (mm)	12
Pathological nodal status	
pN0 (SLNB)	100 (96.1%)
pN0 (ALND)	4 (3.9%)
Free surgical margins (mm)	
≥2-5	31 (29.8%)
>5-10	48 (46.1%)
>10	25 (24.1%)
Histologic type	
Ductal invasive	96 (92.4%)
Lobular invasive	3 (2.9%)
Mucinous invasive	1 (0.9%)
Tubular invasive	2 (1.9%)
Papillary invasive	2 (1.9%)
Histologic grade	
1	67 (64.4%)
2	30 (28.9%)
3	7 (6.7%)
Hormone receptor status	
ER and PR +	90 (86.6%)
ER +, PR -	11 (10.6%)
ER -, PR +	1 (0.9%)
ER and PR -	2 (1.9%)
Endocrine therapy	
Yes	97 (93.3%)
No	7 (6.7%)
Chemotherapy	
Yes	4 (3.9%)
No	100 (96.1%)
Ki67 (%) (n = 69)	
0–20	64(92.8%)
21–40	3(4.4%)
41–60	1(1.4%)
61–80	1(1.4%)
81–100	0 (0%)

ER estrogen receptor, PR progesterone receptor, Ki67 proliferative index.

a Data are n (%) if not otherwise specified.

Before selection for study participation, CT scanning of the operated breast was performed with a slice thickness of 3 mm, in head first-supine position, using CIVCO wing board (Coralville, Iowa, USA) in order to determine if the tumor bed is visually identifiable. Only patients with visible surgical clips and identifiable excision cavity with a cavity visibility score (CVS) of 3–5 were eligible for the study [44]. We used the recommendations of the GEC-ESTRO Breast Working Group for target definition [51]. The clinical target volume (CTV) was defined as the excision cavity plus 20 mm minus the free surgical margins (in mm) in six directions. The CTV expansion was limited to 5 mm beneath the skin surface and at the breast parenchyma/pectoral muscle interface. A uniform, three-dimensional 5 mm margin was added to the CTV to obtain the planning target volume (PTV). For dosimetric reporting, the PTV_EVAL was generated from the PTV limiting the PTV to exclude the first 5 mm tissue under the skin and any lung tissue [23]. The ipsilateral lung, the heart and both breasts were considered as organs at risks. The predefined dose volume and dose homogeneity constraints, registered dosimetric parameters and the method of IG-IMRT were published previously [33]. No respiratory control was used.

The treatment was delivered using three to five isocentric, irregular, non-coplanar photon fields for 3D-CRT, or a step-and-shoot IMRT technique with 5 coplanar photon fields with an average of 30 segments, and with a dose of 9 × 4.1 Gy in two daily fractions (total dose: 36.9 Gy), leaving a minimum of 6-h interval between treatment sessions. Radiotherapy was started within 12 weeks of surgery at 100 (96.2%) patients, when chemotherapy was not given. Anthracycline-based chemotherapy was administered in 4 (3.8%) patients before RT, and APBI was started 3 weeks after the last cycle of chemotherapy. Endocrine therapy (ET) was applied to 97 patients (93.3%), which consisted of aromatase inhibitors in 77 patients (74%) or tamoxifen in 20 women (19.2%) with goserelin acetate being used in 5 cases (4.8%). Six patients (5.8%) refused or stopped ET therapy because of side effects.

**Fig. 1:**
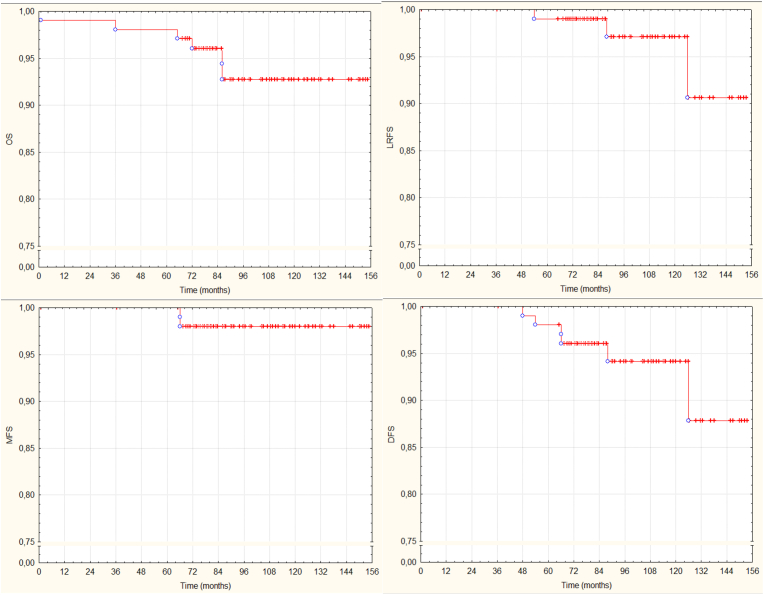
Survival results.

Within 7–14 days after the completion of the RT the acute side effects were recorded. Late side effects (fibrosis and skin side effects) were evaluated every 3 months in the first two years, then every 6 months thereafter, and classified according to the Radiation Therapy Oncology Group/European Society for Therapeutic Radiology and Oncology (RTOG/EORTC) scoring system [45]. Cosmesis were also documented with digital photographs and classified on a 4-point scale (excellent-good-poor-bad) according to Harvard criteria [46]. We reported the patient’s follow up protocol in detail in our previous publications [18,33]. Our primary endpoint was late side effect (telangiectasia/hyperpigmentation, fibrosis, pain), and secondary endpoints were early side effects, survival results, cosmesis, and quality of life. Cumulative OS, LRFS, MFS, DFS results are summarized on Kaplan-Meier curves on Fig. 1. We define local recurrences (LR’s) as the sum of true recurrences/marginal misses, and elsewhere breast failures.

## Results

All treatment plans met the predefined study specific dose constraints for PTV coverage and dose homogeneity, and all patients received the planned nine fractions of APBI. No protocol violation occurred. The average pathologic size of the primary tumor was 12.3 mm (range: 5–30 mm). The average volume of the PTV_EVAL was 152.6 cm^3^ (range: 42.0–460.7 cm^3^). The mean value of the PTV/whole breast volume ratio was 0.17 (range: 0.05–0.40). Mean coverage of the PTV_EVAL by the 95% isodose lines (V95_PTV_EVAL_) and CTV (V95_CTV_) was 99.6% (range: 95–100%) and 99.9% (range: 96–100%), respectively. Mean coverage of the PTV_EVAL by the 90% isodose lines (V90_PTV_EVAL_) and CTV (V90_CTV_) was 99.9% (range: 99.3–100%) and 100% (range: 99.9–100%), respectively. The mean dose covering at least 90% of the PTV_EVAL (D90) was 98.6% of the prescribed dose. Mean maximum dose (D_max_) to the breast was 104.8% (range: 99.2–117%). The mean ipsilateral breast V50 were 48.9% (range: 22–71%) with 3D-CRT and 41.6% (range: 25.8–60.7%) with IG-IMRT. The mean heart dose (MHD) and V5_heart_ was 3.8% (range: 0.5–15.3%) and 17.4% (range: 0–48.3%) for left-sided, and 1.5% (range: 0–4.6%) and 6.45% (range: 0–34.6%) for right-sided lesions, respectively. The mean ipsilateral and contralateral lung dose (MLD) and V10_lung_ was 8.5% (range: 1.5–19%) and 26.6% (range: 2–58%), 1.5% (range: 0.3–6.2%) and 10.5% (range: 0–30.5%), respectively. Without image-guidance, the size of the CTV-PTV margins– according to the assessment of positioning deviations and calculated by the van Herk’s formula – were 9, 10 and 14 mm in the LAT, LONG and VERT directions; whereas with kilovoltage image guidance and "on-line" correction before each fraction, this margin could be reduced to 5, 5 and 6 mm, respectively.

The study median follow-up was 90 months (range: 67–156). So far, three (2.9%) local recurrences (LR) occurred, all in the 3D-CRT arm, the first patient was at 54 months, the second patient at 88 months and the third patient at 126 months after APBI. All three patients underwent salvage mastectomy and they are still alive. The 5 and 7-year cumulative LR rate was 1.1% (95%CI: ±2.1). The mean time to LR was 88.8 months (SD 15.6; range: 54–126). One (0.9%) regional recurrence was observed 48 months after RT, the patient received chemotherapy and regional nodal irradiation. One (0.9%) patient developed contralateral breast cancer 24 months after APBI, and underwent BCS and WBI yielding a 7-year contralateral breast cancer rate of 1.0% [95%CI: ±1.9]). The incidence of first events are listed in Table 2. The 7-year regional relapse (RR) rate was 1.0% (95%CI: ±1.9). Overall, the 7-year loco-regional relapse (LRR) rate was 2.0% (95%CI: ±2.8). Two (1.9%) distant metastases (DM) and 7 (6.7%) secondary tumors were observed. The 7-year actuarial rate of DM was 3.1% (95%CI: +%- 4.3). An overall of 6 (5.8%) patients died, among these 1(0.9%) death was breast cancer related, caused by multiple bone metastases. The 7-year overall survival (OS) was 94.8%. Survival results are summarized in Fig. 1.

**Table 2:** Incidence of first events.

Event	n (%)^a^
Local recurrence	3 (2.9%)
TR/MM	2 (1.9%)
EBF	1 (0.9%)
Regional recurrence	1 (0.9%)
Distant metastasis	2 (1.9%)
Contralateral breast cancer	1(0.9%)
Secondary primary malignancy	7 (6.7%)
Non-breast cancer death	6 (5.8)%

TR/MM true recurrence/marginal miss, EBF elsewhere breast failure.

a Data are n (%).

Stratified analysis of early and late side effects and cosmetic results are listed in Table 3. Acute side effects included grade 1 (G1) and G2 erythema in 54 (51.9%) and 2 (1.9%), G1 parenchymal induration in 43 (41.3%), G1 and G2 pain in 26 (25%) and 2 (1.9%) patients. No ≥ G2 or higher acute side effects occurred. At a median follow-up of 7 years, no patients had any grade 4 toxicities, and one (2.3%) of 44 patients in the 3D-CRT group had grade 3 late subcutaneous tissue toxicity. No ≥ G2 or higher late side effects occurred with IG-IMRT. Late side effects included G1 skin toxicity in 15 (14.4%) patients, and G1, G2, and G3 fibrosis in 26 (25%), 3 (2.9%) and 1 (0.9%) patients, respectively. The cumulative incidence of grade 2 or worse fibrosis was 3.8% at 7 years. Asymptomatic (G1) fat necrosis occurred in 10 (9.6%) patients. G1 pain was observed in 8 patients (7.7%). The cumulative incidence of any late side effects of grade 2 or worse was: 3.8% at 7 years. The rate of excellent/good and fair/poor cosmetic results was 97 (93.3%), and 7 (6.7%), respectively.

**Table 3:** Early and late radiation side effects and cosmetic results.

	3D-CRT (n = 44)	IG-IMRT (n = 60)
Early side effect		
Skin		
G0	11 (25%)	37 (61.7%)
G1	33 (75%)	21 (35%)
G2	0 (0%)	2 (3.3%)
G3	0 (0%)	0 (0%)
Breast parenchyma		
G0	24 (54.5%)	37 (61.7%)
G1	20 (45.5%)	23 (38.3%)
G2-3	0 (0%)	0 (0%)
Pain		
G0	24 (54.5%)	52 (86.7%)
G1	20 (45.5%)	6 (10%)
G2	0 (0%)	2 (3.3%)
G3	0 (0%)	0 (0%)
Late side effects		
Skin	*(n=44)∗*	*(n=60)∗*
G0	37 (84.1%)	52 (86.6%)
G1	7 (15.9%)	8 (13.4%)
G2-3	0 (0%)	0 (0%)
Fibrosis		
G0	20 (45.4%)	54 (90%)
G1	20 (45.4%)	6 (10%)
G2	3 (6.9%)	0 (0%)
G3	1 (2.3%)	0 (0%)
Fat necrosis		
G0	38 (86.4%)	56 (93.3%)
G1	6 (13.6%)	4 (6.7%)
G2-3	0 (0%)	0 (0%)
Pain		
G0	43 (97.7%)	54 (90%)
G1	1 (2.3%)	6 (10%)
G2-3	0 (0%)	0 (0%)
Cosmetic results	*(n=44)*	*(n=60)*
Rated by patients		
Excellent/good	38 (84.1%)	60 (100%)
Fair/poor	6 (15.9%)	0 (0%)
Rated by physicians		
Excellent/good	37 (84.1%)	60 (100%)
Fair/poor	7 (15.9%)	0 (0%)

3D-CRT = three dimensional conformal radiotherapy.

IG-IMRT = image-guided-intensity modulated radiotherapy.

## Discussion

Accelerated partial breast irradiation has been intensively evaluated in multiple phase II and III clinical trials during the last two decades [[11], [12], [13], [14], [15], [16], [17], [18], [19], [20], [21], [22], [23], [24], [25], [26], [27], [28], [29], [30], [31], [32], [33], [34], [35],[38], [39], [40], [41], [42], [43]]. At our institute, between 2006 and 2014, 104 patients were treated with APBI using 3D-CRT or IG-IMRT technique. Our preferred APBI fractionation 36.9 Gy (9 × 4.1 Gy) using twice-a-day was differed to the commonly used 10x3.4–3.85 daily or 5 × 6 Gy every second day fractionation schedule. Our aim was to shorten the treatments for one week (from Monday to Friday) safely, and it was feasible with twice-a-day schedule. Using the linear quadratic model, we calculated an α/β ratio of 3, this prescription was equivalent to 52.4 Gy in a standard 2 Gy fractions. Between the daily fractions a minimum of 6 h passed to recover the normal tissue, thus decrease the toxicity and preserve the good cosmesis however, it has greater biological effect because of incomplete recovery of late reacting normal tissues. According to our results we believe that twice-a-day schedule do not increase the rate of fair/poor cosmetic outcomes.

The 7-year results of our present study are practically identical to the results of contemporary external beam APBI studies (Table 4).

**Table 4:** Local tumor control, late toxicity and cosmetic results of contemporary external beam APBI studies according to treated volume.

Study/Institute	Patient no.	Technique	Fractionation scheme	Median FUP (ys)	LR (%)	G3 toxicity (%)	Excellent/Good cosmesis (%)	Treated volume^a^ (cm^3^)
RAPID (16, 21,39)	1044	3D-CRT/IMRT	10 × 3.85 Gy	8.3	3	4	71	V95: 332
NSAPB B39/RTOG0413(38)	2107	3D-CRT/HDR-iBT/Mammosite	10x 3.85Gy/10x3.4 Gy	10	4.6	7.1	NR	NR
Barcelona (21)	51	3D-CRT	10x3.75 Gy	5	0	0	>75	PTV: 255
William Beaumont II (22)	192	3D-CRT	10 × 3.4 Gy 10x3.85 Gy	4.8	1.6	Fibrosis: 7.5 Skin: 7.6	81	PTV: 269
RTOG 0319 (16, 23)	52	3D-CRT	10x3.85 Gy	5.3	5.8	5.8	57	PTV_EVAL: 177
Tufts University (40)	60	3D-CRT	10 × 3.85 Gy	1.25	0	8.3	82	PTV_EVAL: 296
New York University (17)	98	3D-CRT	5 × 6 Gy	5.3	1	2	89	PTV_EVAL: 162
University Michigan (42)	34	IMRT + ABC	10x3.85 Gy	5	2.9	6.7	73	PTV: 186
Boston (19)	98	3D-CRT	8x4 Gy	5.9	5	NR	NR	NR
Rocky Mountain Cancer Centres (26,28)	136	IMRT	10 × 3.4 Gy; 10x3.85 Gy	4.4	0.7	0	90	NR
Baptist Hospital, Miami (30)	36	IMRT + gating	10 × 3.8 Gy	3.8	3	3	97	PTV: 71
Florence University (28)	260	IMRT	5x6 Gy	10	2.1	0	100	PTV: 150
Current study	104	3DCRT/IG-IMRT	9 × 4.1 Gy	7	2.9	0.9	93	PTV_EVAL: 152

APBI: accelerated partial breast irradiation, 3D-CRT: three-dimensional conformal radiotherapy, IMRT: intensity modulated radiotherapy, IG: image-guided, ABC: active breathing control, NR: Not reported.

a Median values of reported parameters in original publications.

Vicini et al. published the 10-year results of the NSABP B-39/RTOG 0413 phase III study in December 2019 [38]. Overall, 4216 women were randomized between WBI or APBI allowing three alternative techniques (e.g. brachytherapy with single entry device or multicatheter technique, or 3D-CRT). Majority of the patients (71%) in the APBI arm was treated with 3D conformal external beam RT. There were no statistically significant differences in late G3-5 toxicities between the two treatment arms. The absolute difference in the 10-year cumulative incidence of IBTR between APBI and WBI was only 0.7% (4.6% vs 3.9%) with a hazard ratio (HR) of 1.22 (90%CI 0.94–1.58). According to the protocol defined margin to declare the equivalency of APBI and WBI regarding IBTR, the 90% CI of the HR had to lie entirely between 0.667 and 1.5. Therefore, APBI did not meet the criteria for equivalence to WBI. However, as the difference in IBTR was less than 1% at 10 years, the authors concluded that APBI may be an acceptable alternative to WBI for a proportion of women who undergo BCS. It is to be noted that in contrast to other phase III APBI trials high-risk breast cancer patients (young<50 years, premenopausal and node positive patients) were also included in this study, which could lead to a higher IBTR rate. Another limitation of this study is the lack of the human epidermal growth factor receptor 2 (Her2) statuses.

At the same issue of The Lancet Oncology, the long-term results of the RAPID trial was also published by Whelan et al. [39], 2135 women were randomized to receive hypofractionated WBI (42.2 Gy in 16 fractions) or APBI by means of 3D-CRT (38.5 Gy in 10 fractions). After 8.6 years median follow-up, the LR rate was 3% in PBI and 2.8% in WBI arm, but the difference was not statistically significant [HR = 1.27 (90%CI, 0.84–1.91)]. Grade 2 and grade 3 late side effects was 28% and 4% in PBI and 12% and 1% in WBI arm, the 7-year fair and poor cosmetic result was 31% vs 15%, respectively. They conclude that late radiation toxicity may be related to the large treatment volume. In 3D-CRT treatment arm they contoured the CTV as tumor bed + 1 cm in every direction with an additional expansion of another 1 cm to CTV to get PTV. This contouring protocol without image-guidance resulted in large PTV volumes, which probably caused the more serious late side effects and poorer cosmetic results. The V95/whole-breast volume ratio <0.15 was associated with a lower risk of cosmetic deterioration (p = 0.04), but this accounted for only 11% of the patients treated with APBI [48]. They also believe that another component of the increased late radiation toxicity is the accelerated hypofractionated treatment schedule, which was predicted by Yarnold et al., in 2011 [49]. However, no other APBI trial concluded that accelerated treatment schedule led to worse late side effects or cosmetic results, rather than the volume effect which was confirmed by our study results.

The IMPORT-LOW was a multicentric, randomized, controlled phase 3, non-inferiority trial. 2018 women were recruited, 674 patients received 40 Gy hypofractionated WBI, 673 patients received 36 Gy to the whole breast and 40 Gy to the partial breast, and 669 patients 40 Gy to the partial breast only in 15 daily treatment fractions [50]. At a median follow-up of 72.2 months, the 5-year cumulative incidence of IBTR was 1.1% (95% CI 0.5–2.3) of patients in the control group, 0.2% (range: 0.02–1.2) in the reduced-dose group and 0.5% (range: 0.2–1.4) in the partial-breast group. The WBI group showed statistically significant worse adverse effects, including change in breast appearance [p = 0.007 for PBI], and breast harder or firmer [p = 0.002 for RD and p < 0.0001 for PBI] compared with PBI or RD group.

Ten-year results of the Italian prospective randomized study published by the researchers of the Florence University have shown that APBI with IMRT provided 10-year local tumor control equivalent to WBI, 3.7 vs. 2.5% local recurrence rate (p = 0.4) [28]. Concerning acute side effects, the APBI group showed significantly better results any grade (p = 0.0001) and grade 2 or higher (p = 0.0001) cases. No grade 3 toxicity was observed in the APBI group. Erythema was the most frequently observed event for both arms (any grades; 19.9% in APBI, and 66.5% in WBI arm). In terms of late side-effects, only two cases (0.8%) occurred grade 2 toxicity (skin fibrosis), both in the WBI group. No grade 2 or higher late side effects was experienced in the APBI group. In both groups, the cosmetic result was rated as excellent/good for more than 90% of patients. Overall, the APBI group displayed a better cosmetic result and less side effects occurred compared with the WBI group (p = 0.045).

The 5-year results of the GEC-ESTRO phase III APBI trial (using exclusively multicatheter iBT in the investigational arm) were published in 2015 [12]. The cumulative incidence of local recurrence was 1.44% (95% CI 0.51–2.38) with APBI and 0.92% (95% CI 0.12–1.73) with WBI (difference 0.52%, 95% CI – 0.72 to 1.75; p = 0.42). 5-year disease-free survival was 94.4% with WBI and 95.0% with APBI (p = 0.79). The 5-year OS was 95.5% with WBI versus 97.3% with APBI (p = 0.11). The cosmetic results and late side effects were published recently [13]. At a median follow-up of 6.6 years, no patients had any grade 4 toxicities, grade 3 late skin toxicity was <1% in the APBI group and 2% in the WBI group (p = 0.16). No patients in the APBI group and two (<1%) in the WBI group developed grade 3 late subcutaneous tissue toxicity (p = 0.10). After 5 years more than 90% of the patients experienced excellent or good cosmetic results in both treatment groups (91% versus 92%; p = 0.62). Gabani et al. [53] demonstrated with interstitial HDR-BT the significant predictors of fat necrosis were high V_150_ (OR 1.014; P = 0.033), DHI ≤ 0.85 (OR 3.712; P = 0.012), and IRAK>3.400 cGycm^2^/h (OR 2.489; P = 0.019). Rahimi et al. [54] dose escalation S-PBI study presented, fat necrosis developed more frequently with larger PTV (mean 135 cm^3^ vs 95 cm^3^, P = 0.01) and larger breast size(P = 0.02). A cut off point of 124 cm^3^ or higher for PTV volume was predicted as yielding a higher probability of fat necrosis among all dose levels. For an increment of 10 cm^3^ of ipsilateral breast volume there was a 1.4% increase in the odds of fat necrosis development. Similarly, for an increment of 10 cm^3^ of PTV volume, there was a 16% increase in the odds of development of fat necrosis. When dose and volume were evaluated together on logistic regression, both ipsilateral breast volume and PTV volume remained as independent predictors of fat necrosis risk, and dose did not. No other dosimetric parameters yielded a significant correlation to fat necrosis.

Several publications reported higher late toxicity rates and/or adverse cosmetic results following APBI delivered with 3D-CRT or IMRT [16, 20–21, that 40–41]. Rodriguez et al. [21] demonstrated with univariate logistic regression that fibrosis was correlated with the maximum dose (D_max_) within the breast volume, the size of low, and intermediate dose volumes measured as the percentage of the prescription dose (V5% to V50%) as a proportion of the overall breast volume, and the ratio of the PTV_EVAL to the breast volume. Hepel et al. [40] reported 8.3% rate of grade 3–4 subcutaneous fibrosis after a median follow-up of 15 months. Univariate logistic regression analysis revealed that the PTV-Eval/Whole breast volume (WBV) ratio, V_5_/WBV, V_20_/WBV, V_50_/WBV, and V_80_/WBV ratio, and maximal point dose within the breast correlated with Grade 2–4 late subcutaneous fibrosis. A mean V_5_/WBV ratio of 71% was seen in patients without fibrosis compared with 81% for patients with fibrosis. The presence of late subcutaneous fibrosis was the strongest correlate of a fair/poor cosmetic outcome, with an odds ratio of 16 (p < 0.001). The PTV-Eval/WBV ratio, V_5_/WBV ratio, V_20_/WBV ratio, and pathologic volume also correlated with the incidence of fair/poor cosmesis (p = 0.02, p = 0.05, p = 0.04, and p = 0.01, respectively). A mean PTV-Eval/WBV ratio of 18% was seen in patients with excellent/good cosmesis compared with 24% in those with fair/poor cosmesis. Jagsi et al. [40] retrospective analysis demonstrated between patients with acceptable and unacceptable cosmesis revealed that the mean proportion of breast volume receiving a minimum of 100% of the prescribed dose (38.5 Gy; V100) was lower in patients with acceptable cosmesis (16% vs. 23.5%). The mean percentage of the breast volume receiving a minimum of 50% of the prescribed dose (19.25 Gy; V50) was also smaller (35.5% vs. 45.4%) in the acceptable cosmesis, group. They also find that tumor location was significantly different between patients with unacceptable cosmesis (80% with inner/central tumors) and those with acceptable cosmesis (27% with inner/central tumors). In Our study the PTV-Eval/WBV ratio was 0.17 (range: 0.05–0.40), mean ipsilateral breast V50 were 48.9% (range: 22–71%) with 3D-CRT and 41.6% (range: 25.8–60.7%) with IG-IMRT. Our primary dose constrains prescribed for ipsilateral breast have been met V50 ≤ 60%. The only patient who had V50 > 60% (71%), Gr2 fibrosis observed with fair cosmetic result, however all the other patients who had Gr2 fibrosis or fair cosmetic results the V50 value were much less than 60%.

Shah et al. [22] also reported a 7.5% rate of grade 3–4 fibrosis after 5 years. In these studies, the increased rate of late toxicity was probably due to the large irradiated target volume since the PTV values were in the range of 175–330 cm^3^. In an effort to minimize the volume of the PTV, we performed image guidance before the delivery of each fraction. Using IG-IMRT, CTV-PTV margins could be reduced to 5 mm yielding a limited mean target volume (PTV_EVAL) of 152.6 cm^3^.

For decades conventionally fractionated WBI was the gold standard treatment over 5–7 weeks. The Start trials showed that mildly hypofractionated WBI over 3 weeks is safe and patient-friendly in terms of toxicity and treatment time shortening. In 2006 only our APBI trial offered one-week treatment schedule (Table 4). In the Florence trial (28) a total dose of 30 Gy in 5 non-consecutive once-daily fractions was given over two weeks, the same as the prone APBI trial in New York (17). Nowadays, according to the 10-year results of FAST-FORWARD trial WBI was also given in 5 fractions, over 1 week, with 5.2 Gy daily dose, as a new standard for adjuvant radiotherapy of the breast [55]. Further ongoing phase II-III trials (PRECISION, DBCG RT NATURAL, EUROPA) investigating the treatments de-escalation or RT omission, aim to identify the best precision strategy for low-risk patients.

## Conclusions

APBI delivered by 3D-CRT and IG-IMRT are technically feasible and seems to be a safe adjuvant therapy for selected early-stage breast cancer patients treated with BCS. Based on our experience, image guidance performed before each radiation fraction is necessary for the reduction of the target volume to avoid severe late side effects. According to our 7-year results APBI using external beam 3D-CRT and IG-IMRT is well tolerated by the patients, late side effects are mild and cosmetic results are excellent. According to long-term data from prospective randomized trials APBI with multicatheter iBT or IG-IMRT are acceptable alternatives to WBI after BCS. Accelerated hypofractionated treatment schedule did not increase the rate of higher late toxicity or worse cosmetic results.

## Statement

I state here, that ethical or animal rights approval was not required.

## Declaration of competing interest

N. Mészáros, T. Major, G. Stelczer, L. Jánváry, Z. Zaka, D. Pukancsik, Z. Takácsi-Nagy, J. Fodor, and C. Polgár state that there are no conflicts of interest.
